# Gut Microbiotic Features Aiding the Diagnosis of Acute Ischemic Stroke

**DOI:** 10.3389/fcimb.2020.587284

**Published:** 2020-12-21

**Authors:** Lei Xiang, Yanfeng Lou, Lingyu Liu, Yuanling Liu, Weizheng Zhang, Jianxin Deng, Yubin Guan, Miaoqin She, Xinchao You, Minqi Liu, Hongwei Li, Xiaosong Xu, Fang Liu, Xiangsheng Cai

**Affiliations:** ^1^Department of Integrative Chinese and Western Medicine, The First Affiliated Hospital of Guangdong Pharmaceutical University, Guangzhou, China; ^2^Department of Dermatology, Jinling Hospital, Southern Medical University, Nanjing, China; ^3^School of Traditional Chinese Medicine, Southern Medical University, Guangzhou, China; ^4^Administrative Department, Guangdong Province Hospital for Women and Children Healthcare, Guangzhou, China; ^5^Clinical Laboratory, The Second Affiliated Hospital of Guangzhou University of Chinese Medicine, Guangzhou, China; ^6^Department of Endocrinology, Shenzhen Second People’s Hospital, The First Affiliated Hospital of Shenzhen University, Health Science Center of Shenzhen University, Shenzhen, China; ^7^Clinical Laboratory, The First Affiliated Hospital of Guangdong Pharmaceutical University, Guangzhou, China; ^8^Research Section, The First Affiliated Hospital of Guangdong Pharmaceutical University, Guangzhou, China; ^9^Guangzhou Institute of Biomedicine and Health, Chinese Academy of Sciences, Guangzhou, China; ^10^Institute of Biotherapy, Southern Medical University, Guangzhou, China; ^11^Center for Medical Experiments, University of Chinese Academy of Science-Shenzhen Hospital, Shenzhen, China

**Keywords:** lacunar infarction, post-ischemic stroke, gut microbiota, acute ischemic infarction, non-lacunar acute ischemic infarction, random forest model

## Abstract

Increasing evidence suggests that features of the gut microbiota correlate with ischemic stroke. However, the specific characteristics of the gut microbiota in patients suffering different types of ischemic stroke, or recovering from such strokes, have rarely been studied, and potential microbiotic predictors of different types of stroke have seldom been analyzed. We subjected fecal specimens from patients with lacunar or non-lacunar acute ischemic infarctions, and those recovering from such strokes, to bacterial 16S rRNA sequencing and compared the results to those of healthy volunteers. We identified microbial markers of different types of ischemic stroke and verified that these were of diagnostic utility. Patients with two types of ischemic stroke, and those recovering from ischemic stroke, exhibited significant shifts in microbiotic diversities compared to healthy subjects. Cluster of Orthologous Groups of Proteins (COG) and Kyoto Encyclopedia of Genes and Genomes (KEGG) pathway analyses revealed reduced metabolic and transport-related pathway activities in ischemic stroke patients. We performed fivefold cross-validation using a Random Forest model to identify two optimal bacterial species (operational taxonomic units; OTUs) serving as markers of lacunar infarction; these were *Lachnospiraceae* (OTU_45) and *Bacteroides* (OTU_4), and the areas under the receiver operating characteristic curves (AUCs under the ROCs) were 0.881 and 0.872 respectively. In terms of non-lacunar acute ischemic infarction detection, the two optimal species were *Bilophila* (OTU_330) and *Lachnospiraceae* (OTU_338); the AUCs under the ROCs were 0.985 and 0.929 respectively. In post-ischemic stroke patients, the three optimal species were *Pseudomonas* (OTU_35), *Sphingomonadaceae* (OTU_303), and *Akkermansia* (OTU_9); the AUCs under the ROCs were 1, 0.897, and 0.846 respectively. Notably, the gut microbial markers were of considerable value for utility when diagnosing lacunar infarction, non-lacunar acute ischemic infarction, and post-ischemic stroke. This study is the first to characterize the gut microbiotic profiles of patients with lacunar or non-lacunar, acute ischemic strokes, and those recovering from stroke, and to identify microbiotic predictors of such strokes.

## Introduction

Ischemic stroke is a major cause of death and severe neurological disability; global post-stroke mortality is 39% (Wang et al., 2017). Unhealthy living habits, excessive stress, and aging have increased the global incidence of cerebral ischemia. Acute ischemic infarction is the most common type of stroke, associated with high rates of death and disability (Paciaroni et al., 2008). Acute ischemic infarction subtypes were classified based on clinical data using the Oxfordshire Community Stroke Project (OCSP) classification scheme as total anterior circulation infarcts [TACIs], partial anterior circulation infarcts [PACIs], posterior circulation infarcts, and lacunar infarcts [LACIs]) (Sandset et al., 2015). Lacunar infarctions are small (<2-cm-diameter) infarctions that account for approximately 25% of all strokes; LIs differ pathologically from large artery strokes, including TACI, PACI, and POCI (Das et al., 2019). Thus, some studies divide acute ischemic infarctions into lacunar and non-lacunar acute ischemic infarctions (Regenhardt et al., 2019; Zhou et al., 2020). Post-ischemic stroke patients have experienced acute ischemic stroke and often deal with severe sequelae including cognitive and anxio-depressive disorders, fatigue, and restricted participation in daily life (Zhang et al., 2018). Though the progress were made in the treatments of stroke, the prognosis is poor, most ischemic stroke patients are diagnosed at advanced stages causing high risks of disability and mortality. Therefore, early diagnostic measures of ischemic stroke need to be explored so as to greatly improve the management of the disease.

Gut microbiotic alterations are newly identified risk factors for stroke; patients exhibit significant dysbiosis in terms of both microbial diversity and composition (Yin et al., 2015). A recent study found that ischemic stroke altered the gut microbiome, reduced microbiome diversity, and increased the immune response (Singh and Roth, 2016). Ischemic stroke triggered maladjustment of the mouse cecal microbiota within 72 h (Houlden et al., 2016). Thus, the gut microbiota was presumed to play roles in stroke initiation and development (Zhang et al., 2015; Langdon et al., 2016). Moreover, many neurological disorders (Alzheimer’s and Parkinson’s diseases, depression, and multiple sclerosis) are commonly accompanied by gastrointestinal symptoms (McDonald and Cervenka, 2018). Therefore, the microbiota-brain axis is proposed and is thought to constitute a bidirectional informational network linking the gut microbiota and the brain (Wang and Wang, 2016). Despite extensive analysis of the link between the gut microbiome and stroke, the microbiotic features of different types of stroke have rarely been studied. Moreover, it remains unclear whether the overall gut microbiota, or only specific bacterial species, affect stroke outcomes; no report has described microbiotic or bacterial predictors of stroke. Here, we describe changes in gut microbiotic species in patients with lacunar or non-lacunar, acute ischemic strokes, and those recovering from acute ischemic strokes, and we identify potential bacterial predictors of such strokes.

## Materials and Methods

### Samples

This study was approved by the Ethics Committee of the First Affiliated Hospital of Guangdong Pharmaceutical University. All participants signed written informed consent prior to enrolment. Fecal samples were collected from 16 healthy volunteers (N group, *n* = 16); 20 patients with ischemic stroke: lacunar infarction (LI group, *n* = 10), non-lacunar acute ischemic infarction (AI group, *n* = 10); and 10 post-ischemic stroke patients who had undergone 15 days of treatment after acute ischemic stroke (PI group, *n* = 10). All of age, sex and the body mass index were comparable among the groups (**Table 1**; all *P*>0.05). The enrolled stroke patients were satisfied the clinical diagnostic criteria and were confirmed by magnetic resonance imaging (MRI) or brain computed tomography (CT). The exclusion criteria included respiratory or renal failure, a recent cardiac event, an immune system condition, intestinal disease or severe liver dysfunction, or the use of probiotics or antibiotics within 1 month prior to admission. The healthy volunteers who had not taken antibiotics, probiotics, or possibly confounding drugs within the prior 1 month were enrolled.

**Table 1:** Characteristics of each group.

Group	N	LI	AI	PI	*P*-value
Gender (M/F)	7/9	4/6	5/5	5/5	0.9592
Age, year	71 (61–81)	72 (57–89)	73 (59–87)	73 (60–83)	0.8049
BMI (kg/m^2^)	24.1(20.4–27.2)	24.8(21.2–28.4)	25.1(21.8–28.1)	24.9 (20.2–28.6)	0.6914

### Extraction, Amplification, and Sequencing of Bacterial Genomic DNA From Fecal Samples

Fresh fecal samples were collected into Eppendorf tubes and frozen at −80°C. Bacterial genomic DNA was extracted using QIAamp DNA Stool Mini Kits according to the manufacturer’s instructions. The V3 variable regions of 16S rDNA genes were subjected to PCR amplification as described previously (Muyzer et al., 1993; Fan et al., 2016): predenaturation at 94°C for 5 min; 30 cycles of denaturation at 94°C for 30 s, annealing at 55°C for 30 s, extension at 72°C for 30 s; and a final extension at 72°C for 10 min. Fragments were purified *via* agarose gel electrophoresis and re-electrophoresed to determine DNA concentrations using the Qubit 2 method. The mixed products were sent to our Sequencing Institute.

### OTUs, Abundances, and Complexity Analysis

The data were filtered by Mothur software and clustered into operational taxonomic units (OTUs; species) at a similarity level of 97% using Quantitative Insights into Microbial Ecology (QIIMEv) software ver. 1.80 (Houlden et al., 2016). All OTUs in the discovery and validation sets were recorded. Gut microbiota alpha diversity indices (the Observed Species, Chao, ACE, Shannon, and Simpson indices) were calculated by Mothur ver. 1.31.2 software and dilution curves and box diagrams created with the aid of R software ver. 3.1.1. Beta diversity analysis was used to compare microbiotic species diversities. Principal component analysis (PCA) was performed with the aid of the Ade4 R package; this revealed similarities among relative microbial abundances. To compare microbial compositions among samples, principal coordinate analysis (PCoA) and nonmetric multidimensional scaling (NMDS) were applied, and the Bray-Curtis distances were used to generate two-dimensional plots.

### Species Composition

The microbial compositions after grouping were compared at the phylum, class, and genus levels. Beta diversity heatmaps were plotted using the NMF package of R. The Bray-Curtis distances lay between 0 and 1; higher values indicated greater between-sample differences. To identify bacteria that differed significantly among groups, the taxon summaries were reformatted and linear discriminant analysis effect sizes (LEfSes) determined with the aid of the Huttenhower Lab Galaxy Server. In this setting, the Kruskal-Wallis sum-rank test (α = 0.05) was initially used to detect taxa exhibiting significantly different abundances. The false discovery rate (FDR) was the corrected *P*-value; an FDR<0.05 was considered significant. Biological consistency was then investigated *via* pairwise testing among subclasses using the Wilcoxon rank-sum test. Finally, linear discriminant analysis (LDA) was employed to estimate the effect sizes of differentially abundant gut microbiota. The logarithmic LDA threshold for discrimination was 2.0. The Clusters of Orthologous Group (COG) annotations were determined using PICRUSt2 software. The affected Kyoto Encyclopedia of Genes and Genomes (KEGG) Pathways were identified using the KEGG Automatic Annotation Server (KAAS).

### Statistical Analyses

The Wilcoxon rank-sum test (two groups) or Kruskal-Wallis test (multiple groups) of R ver. 3.0.3 software were used for inter-group comparisons. The t- test, chi-squared test, and the Spearman rank correlation analysis of SPSS ver. 19.0 were employed as appropriate. A *P*-value <0.05 was considered to reflect statistical significance.

A species-based classifier was trained using the Random Forest package of R. The model was employed for fivefold cross-validation of the relative species abundance profile. Case probabilities were calculated by drawing receiver operating characteristic (ROC) curves.

## Results

### OTU Distributions

**Figure 1:**
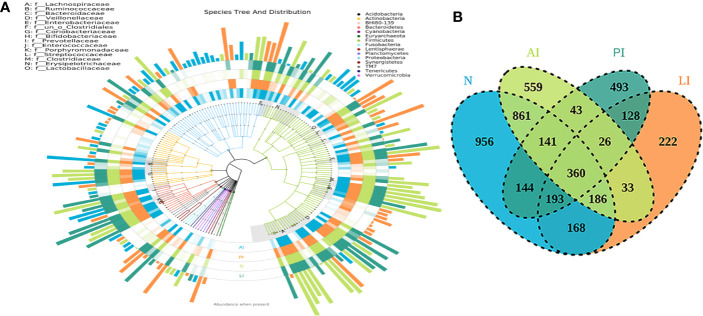
**(A)** The species trees and distributions of gut microbial communities. **(B)** A Venn diagram showing OTU similarities and differences among the groups.

The OTUs annotated for subsequent analyses included 22 phyla, 116 families, and 217 genera of gut microbes inferred *via* V4 amplicon sequences (39 to 297 base pairs); the among-sample similarity was 97% (**Figure 1A**). The total OTUs of the LI, PI, and AI groups (at the 97% similarity level) were 1,094, 1,528, and 1,650 respectively, and 2,053 for the control group. OTUs shared by the stroke and control groups numbered 360, as revealed by a Venn diagram (**Figure 1B**).

### Alpha and Beta Diversities

**Figure 2:**
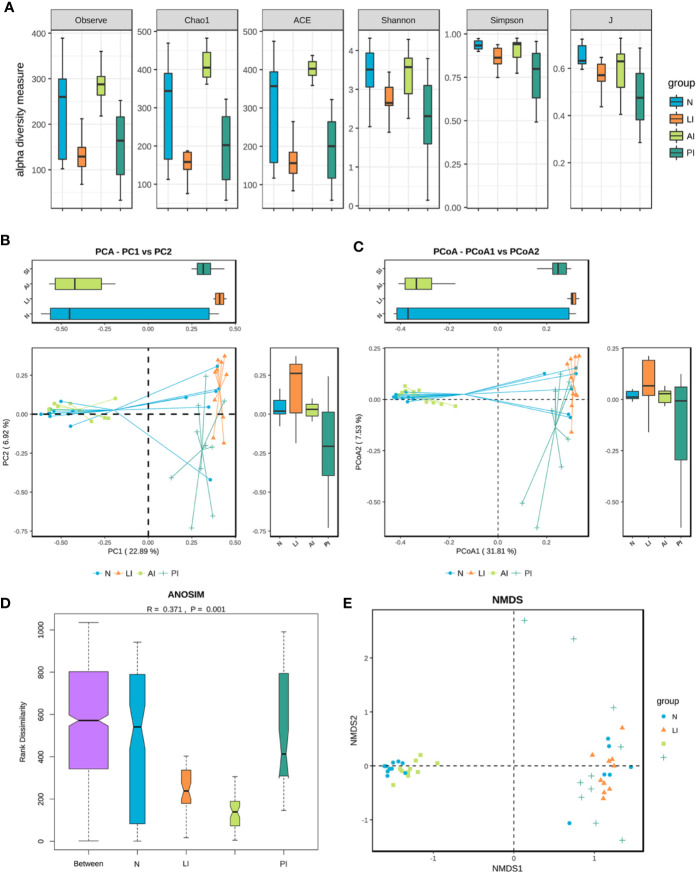
**(A)** Gut microbiotic alpha and beta diversity indices of ischemic stroke patients. The Observed_species, Chao1, ACE, Shannon, Simpson and J index values. **(B)** PCA scores based on the relative abundances of OTUs (at the 97% similarity level). **(C)** PCoA analysis based on weighted Unifrac distances. **(D)** Analysis of similarities. **(E)** Non-metric multi-dimensional scaling. Each dot represents a sample; the group is identified in the legend. In the graphs, closer samples are more similar.

In terms of alpha diversity, the chao1 richness index of the AI group differed significantly from those of the LI, PI, and control groups (61.3 P = 0.0; 51.9 P = 0.0002; and 28.6375 P = 0.0272). The Observed_species index of the AI group differed significantly from those of the LI and PI groups (52.75 P = 0.005; 45.95 P = 0.0015). The Shannon and Simpson indices of the PI group differed significantly from those of the AI and control groups (Shannon 35.8 P = 0.0369, 36.3375 P = 0.0369; Simpson 35 P = 0.0386 40.15 P = 0.0216) (**Figure 2A**). The ACE index of the AI group differed significantly from those of the LI, PI, and control groups (60.4 P = 0.0; 51.6 P = 0.0002; 26.8125 P = 0.0381). PCA and PCoA indicated that the AI microbiome differed significantly from those of the LI and PI groups (which were clustered), and was comparable to that of healthy controls (**Figures 2B, C**). Analysis of similarities (ANOSIM) indicated that the gut microbiotic structure differed significantly among the groups (ANOSIM, *r* = 0.371, *P* = 0.001) (**Figure 2D**). NMDS analysis based on the Bray-Curtis distances between microbial genera revealed significant differences between AI patients and the other three groups (**Figure 2E**).

### Taxonomy

**Figure 3:**
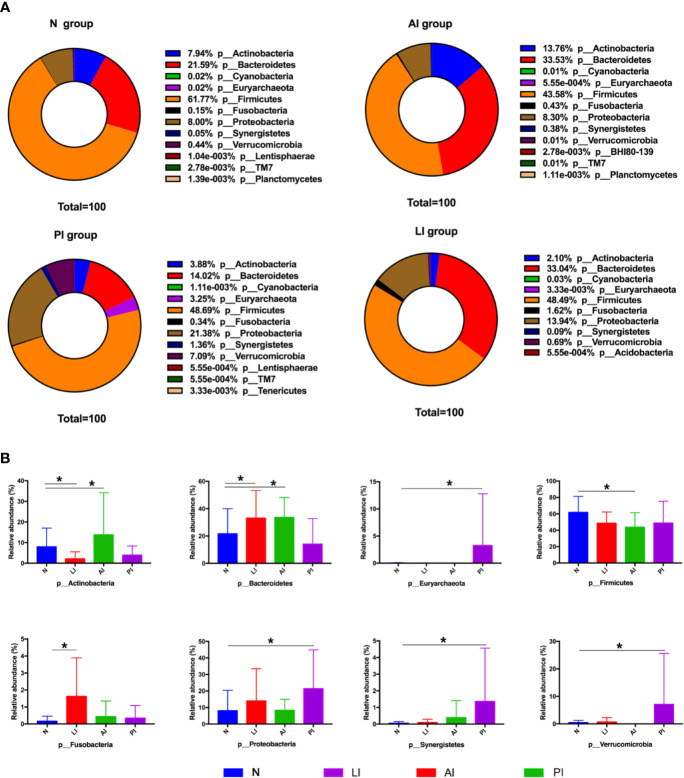
**(A)** Taxonomic profiling assigned most OTUs to *Firmicutes*, *Bacteroidetes*, *Actinobacteria*, *Proteobacteria*, *Verrucomicrobia*, and *Fusobacteria* at the phylum level. **(B)** Among-group differences at the phylum level. *P < 0.05.

The gut microbiota differed among the groups at the phylum, family, and genus level. At the phylum level, *Firmicutes*, *Bacteroidetes*, *Proteobacteria*, and *Actinobacteria* were the most common phyla of the four groups, comprising 99.73% of all gut bacteria in the AI group; the figures for the PI, LI, and control groups were 87.97, 97.57, and 99.3% respectively (**Figures 3A, B**). All stroke patients exhibited fewer *Firmicutes* than controls (*P*<0.05). LI group contained more *Cyanobacteria* and *Fusobacteria* than the other groups. AI group exhibited markedly higher *Actinobacteria* levels (*P*<0.05) than the other groups. *Verrucomicrobia, Synergistetes*, and *Proteobacteria* were significantly enriched in the PI compared to the other groups (*P*<0.05). The ratio of *Firmicutes : Bacteroidetes* was lower in AI and LI groups but higher in PI group compared to healthy controls (1.30 in AI group, 3.47 in PI group, 1.47 in LI group, and 2.86 in control group).

**Figure 4:**
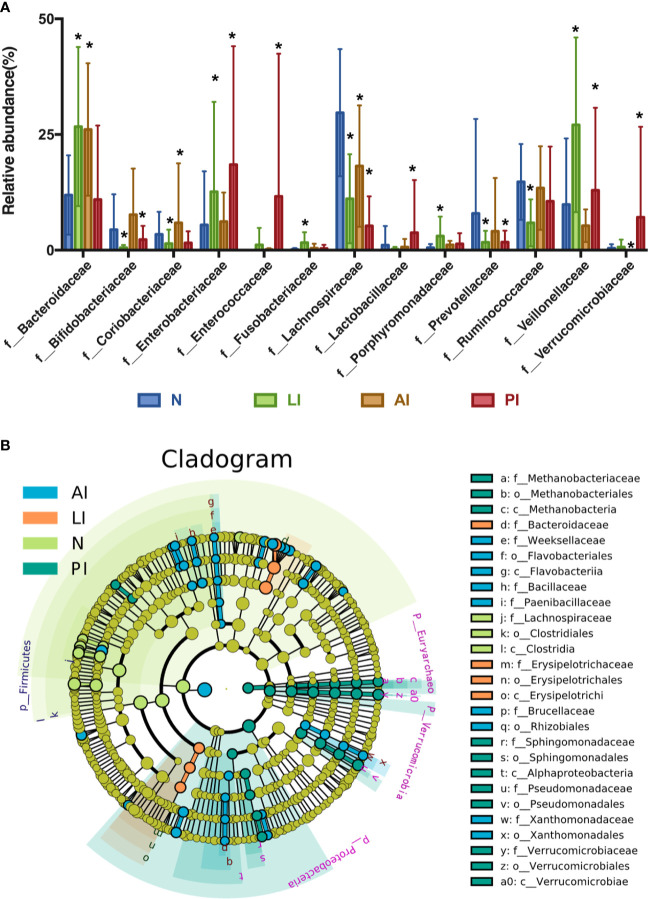
**(A)** The most abundant microbiotic components (at the family level) of each group. *P < 0.05. **(B)** LEfSE analysis reveals bacterial taxa, the levels of which differ significantly among groups.

At the family level, individual variations were more marked; the dominant taxa differed among the groups (**Figure 4A**). Of all families identified, 74, 60, 51, and 75 of the dominant families were present in AI, PI, LI, and control groups respectively. *Bacteroidaceae*, *Lachnospiraceae*, *Ruminococcaceae*, *Bifidobacteriaceae*, *Enterobacteriaceae*, *Coriobacteriaceae*, *Veillonellaceae*, and *Prevotellaceae* were the eight most abundant microbiotic components in all groups. *Bacteroidaceae* levels were significantly higher in AI and LI groups (*P*<0.05) compared to the other groups. *Bifidobacteriaceae* and *Coriobacteriaceae* were most abundant in AI group (*P*<0.05). *Veillonellaceae* levels were highest in the LI group (*P*<0.05). *Enterobacteriaceae*, *Enterococcaceae*, *Lactobacillaceae*, and *Verrucomicrobiaceae* levels were highest in PI group (*P*<0.05). *Lachnospiraceae*, *Ruminococcaceae*, and *Prevotellaceae* levels were maximal in control group (*P*<0.05).

LEfSE analysis was used to identify bacterial taxa, the levels of which differed significantly among groups (**Figure 4B**). *Weeksellaceae*, *Bacillaceae*, *Paenibaciiaceae*, *Brucellaceae*, and *Xanthomnadaceae* were significantly more abundant in AI group. *Bacteroidaceae* and *Erysipelotrichaceae* levels were highest in LI group. *Methanobacteriaceae*, *Sphingomonadaceae*, *Pseudomonadaceae*, and *Verrucomicrobiaceae* levels were maximal in PI group. *Lactobacillaceae* was most abundant in control group.

**Figure 5:**
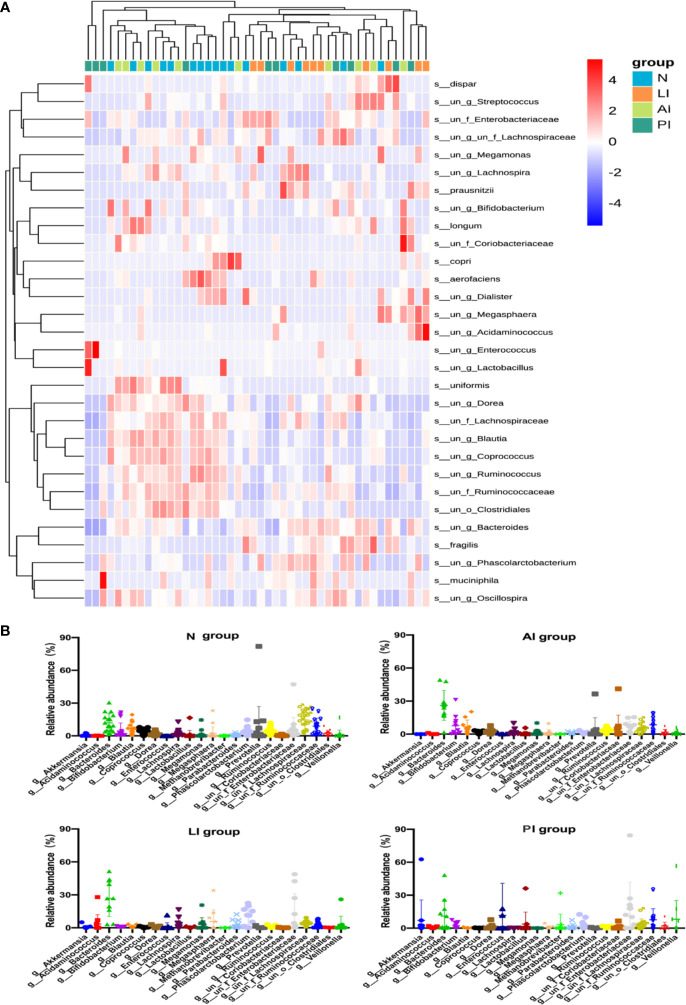
**(A)** A heat map showing the relative total abundances of the top 30 species, and the relative abundances of all species, at the genus level. **(B)** A taxonomic summary of the gut microbiota of each group at the genus level. Starring (*) indicates a significant difference between two groups.

The genus-level compositions were more complex; the levels of many genera differed significantly among the groups (**Figures 5A, B**). In control group, *Lachnospiraceae*, *Bacteroides*, *Ruminococcaceae*, *Prevotella*, *Blautia, Enterobacteriaceae, Bifidobacterium*, and *Ruminococcus*, were the eight principal genera (of 132). In LI group, the eight predominated genera (of 100) were *Bacteroides, Enterobacteriaceae, Phascolarctobacterium, Megasphaera, Lachnospiraceae*, *Acidaminococcus*, *Lachnospira*, and *Parabacteroides*. In AI group, the eight common genera (of 136) were *Bacteroides*, *Ruminococcaceae*, *Bifidobacterium*, *Blautia*, *Enterobacteriaceae*, *Lachnospiraceae*, *Coriobacteriaceae*, and *Prevotella*. In PI group, *Enterobacteriaceae*, *Enterococcus*, *Bacteroides*, *Veillonella*, *Ruminococcaceae*, *Akkermansia*, *Lactobacillus*, and *Lachnospiraceae* were eight predominated genera (of 121).

### Functional Profiling of the Gut Microbiome

**Figure 6:**
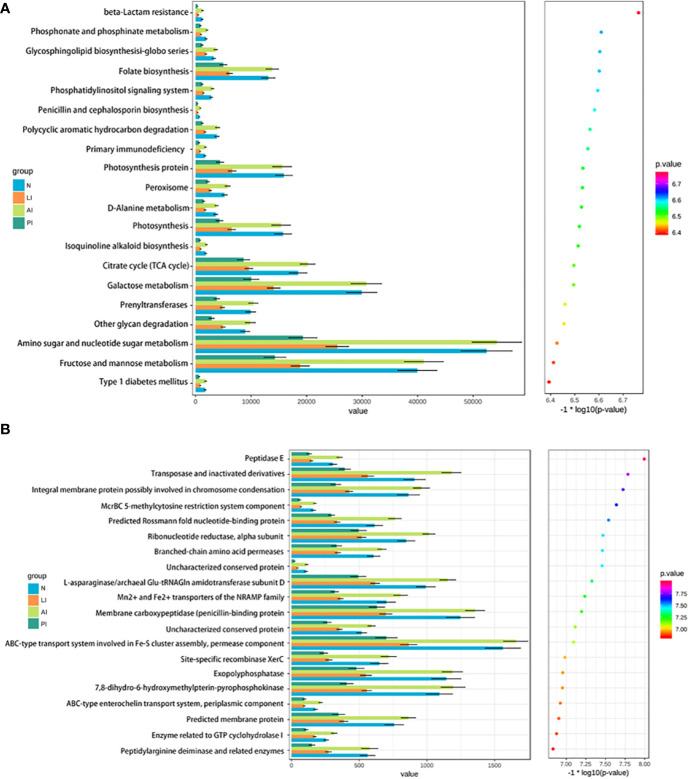
KEGG pathways **(A)** and COG categories **(B)** that was less frequent in stroke groups than healthy controls.

We compared the COG and KEGG pathways among the groups; we sought differences in microbiotic functions between stroke patients and controls. **Figure 6A** shows that nine functional KEGG pathways were highly enriched in both AI patients and healthy controls. These included the pathways of folate biosynthesis, photosynthesis, peroxisomal action, the citrate cycle (TCA cycle), galactose metabolism, phenyltransferase, other glycan degradation, amino sugar and nucleotide sugar metabolism, and fructose and mannose metabolism. **Figure 6B** shows that 12 COG categories were highly enriched in AI patients and healthy subjects, including transposase (and its inactivated derivatives), the predicted Rossmann fold nucleotide-binding protein, the ribonucleotide reductase alpha subunit, the l-asparaginase/archaeal Glu-tRNAGln amidotransferase subunit D, the Mn^2+^ and Fe^2+^ transporters of the NRAMP family, the ABC-type transport system involved in Fe-S assembly, a permease component, the site-specific recombinase XerC, an exopolyphosphatase, 7,8-dihydro-6-hydroxymethylpterin-pyrophosphokinase, a predicted membrane protein, an enzyme related to GTP cyclohydrolase I, and a peptidylarginine deaminase and related enzymes. The transposase (and inactivated derivatives), predicted Rossmann fold nucleotide-binding protein, ribonucleotide reductase alpha subunit, and L-asparaginase/archaeal Glu-tRNAGln amidotransferase subunit D were significantly more abundant in the AI than the LI or PI groups (all *P*<0.05).

### Random Forest Identification of Ischemic Stroke Markers

**Figure 7:**
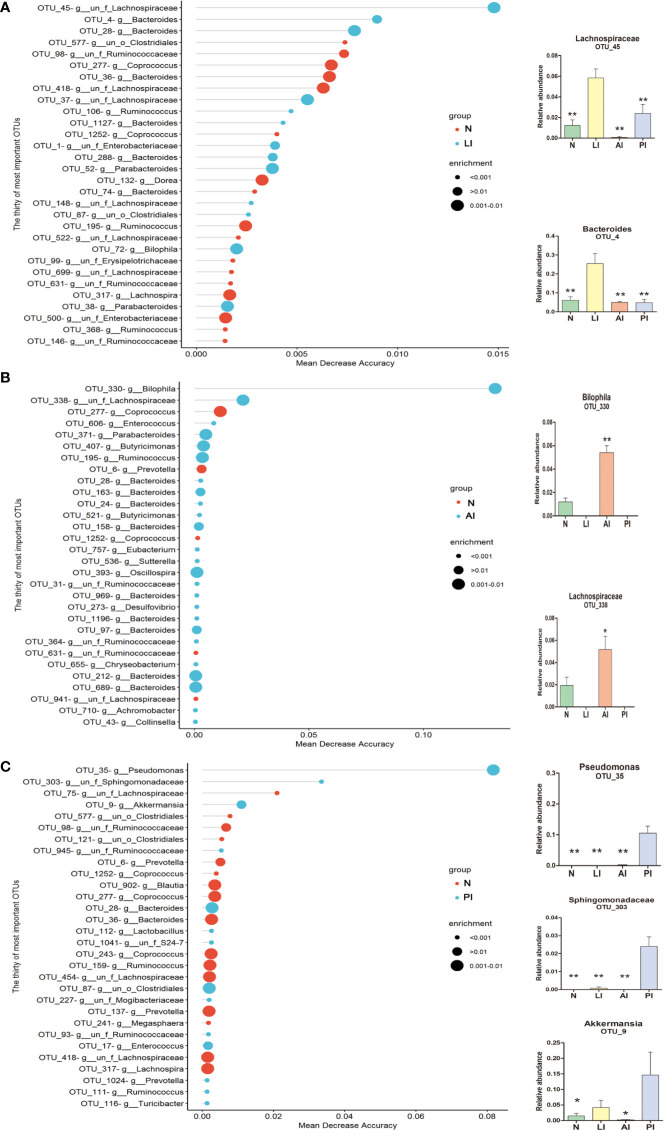
OTU-based markers of ischemic stroke identified by the Random Forest model. **(A)** lacunar infarction. **p < 0.01 compared with the LI group. **(B)** non-lacunar acute ischemic infarction. *p < 0.05 and **p < 0.01 compared with the AI group. **(C)** post-ischemic stroke. *p < 0.05 and **p < 0.01 compared with the PI group.

**Figure 8:**
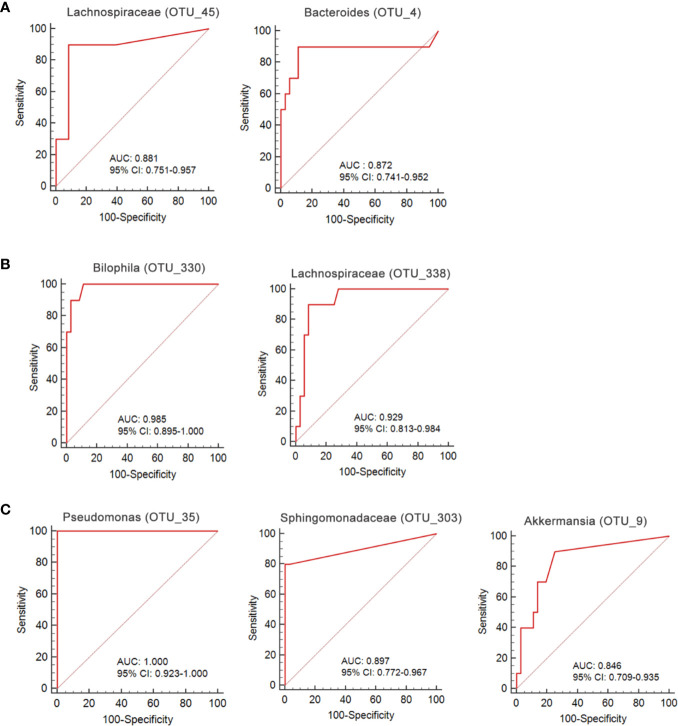
ROC curves for the OTU-based diagnostic biomarkers of ischemic stroke. **(A)** lacunar infarction; **(B)** non-lacunar acute ischemic infarction; **(C)** post-ischemic stroke.

We used a Random Forest model to identify microbial profiles that optimally differentiated the ischemic stroke and healthy groups; the Random Forest is a robust machine-learning technique that can handle nonlinear relationships and dependencies among microbiotic features. We performed fivefold cross-validation five times using a validation set (**Figures 7A–C**). Two species afforded optimal LI detection: *Lachnospiraceae* (OTU_45) and *Bacteroides* (OTU_4); the areas under the receiver operating characteristic curves (AUCs under the ROCs) were 0.881 and 0.872 respectively (**Figure 8A**, **Table 2**). Two species optimally detected AI: *Bilophila* (OTU_330) and *Lachnospiraceae* (OTU_338); the AUCs under the ROCs were 0.985 and 0.929 respectively (**Figure 8B**, **Table 2**). Three species optimally detected PI: *Pseudomonas* (OTU_35), *Sphingomonadaceae* (OTU_303), and *Akkermansia* (OTU_9); the AUCs under the ROCs were 1, 0.897, and 0.846 respectively (**Figure 8C**, **Table 2**).

**Table 2:** Diagnostic values of significant gut microbiota for acute ischemic stroke.

Disease	Species	AUC	SE	Sig.	95% C.I. for AUC
LI	*Lachnospiraceae* (OTU_45)	0.881	0.071	<0.01	0.751~0.957
	*Bacteroides* (OTU_4)	0.872	0.096	<0.01	0.741~0.952
AI	*Bilophila* (OTU_330)	0.985	0.014	<0.01	0.895~1.000
	*Lachnospiraceae* (OTU_338)	0.929	0.039	< 0.01	0.813~0.984
PI	*Pseudomonas* (OTU_35)	1.000	0.000	<0.01	0.923~1.000
	*Sphingomonadaceae* (OTU_303)	0.897	0.069	<0.01	0.772~0.967
	*Akkermansia* (OTU_9)	0.846	0.068	<0.01	0.709~0.935

## Discussion

This is the first study to explore the characteristics of the gut microbiota in patients suffering from different types of acute ischemic stroke, or recovering from such strokes. The gastrointestinal tract is a major organ of the immune system, containing over 70% of all immune cells and the largest population of macrophages in the human body. The composition of the gut microbiota depends on many factors, including lifestyle, diet, metabolism, antibiotic use, and hygiene; all are also closely associated with ischemic stroke (Bibbò et al., 2016). Several reports have described intestinal dysfunction and gut dysbiosis after stroke, highlighting the delicate interplay between the brain, gut, and microbiome after acute brain injury (Benakis et al., 2016; Singh and Roth, 2016; Zhao et al., 2018). The “brain-gut axis” is considered to facilitate bidirectional communication between the central nervous system and the gastrointestinal tract (Khlevner et al., 2018). Although many studies have confirmed the relationship between stroke and gut microbiome, the microbiotic status of patients differing in terms of stroke status has not been explored.

In this study, the phylum *Firmicutes* was less abundant in three stroke groups than the control group. The genera of butyrate-producing bacteria (e.g., *Lachnospiraceae*) were less common in the stroke groups. Increasing evidence indicates that the short-chain fatty acid (SCFA) metabolites (especially butyric acid) of gut bacteria are key signaling molecules (Stilling et al., 2016). Butyric acid supplementation significantly reduced intestinal leakage in patients with cerebral ischemic strokes and reduced levels of butyrate-producing bacteria may trigger stroke (Singh and Roth, 2016). Transplantation of fecal bacteria that produce SCFAs, and butyric acid supplementation, may effectively treat cerebral ischemic stroke.

AI and LI groups exhibited lower *Firmicutes*: *Bacteroidetes* ratios, and a higher ratio in PI group, than healthy controls. A reduced ratio reflects dysbiotic gastrointestinal tract metabolism, associated with low levels of circulating SCFAs, compromising systemic immunity and triggering systemic inflammation. It may be possible to reduce the risk of stroke, and prevent stroke progression and complications, by manipulating the ratio (Eckburg et al., 2005).

Each stroke group exhibited unique microbiotic features. *Fusobacteria* and *Cyanobacteria* were most abundant in LI group. Notably, LEfSE analysis showed that *Bacteroidaceae* and *Erysipelotrichaceae* were more abundant in LI than other groups. To the best of our knowledge, this is the first such report. The opportunistic pathogens of the *Veillonellaceae* were more abundant in LI group, as previously reported by Zeng et al., (2019). Lacunar infarcts are thought to be primarily attributable to intracranial, small vessel disease. Endothelial dysfunction may play important roles in the pathogenesis and progression of such disease (Regenhardt et al., 2018). *Ruminococcaceae* levels were dramatically decreased in LI group, and beneficial bacteria such as *Meganonas* enriched. Emerging evidence indicates that microbiotic interventions may enhance the efficacy of therapy and ameliorate toxicity; the gut microbiome performs several vital functions including vitamin production and dietary metabolism, and protects against gut pathogen expansion and systemic infiltration.

AI group exhibited markedly higher levels of *Actinobacteria* than other groups. A mouse study found that the gut microbiota affected stroke progression by acting on the immune system (Benakis et al., 2016). The opportunistic pathogens *Bacteroidaceae*, and lactate-producing bacteria (e.g., *Bifidobacterium*), were enriched in AI group. The serum trimethylamine N-oxide (TMAO) concentration is positively associated with the first stroke in hypertensive patients. TMAO is produced when hepatic flavin mono-oxygenases act on trimethylamine, a waste product of gut microbes (Zeisel and Warrier, 2017). *Ruminococcaceae*, *Bifidobacteriaceae*, and *Coriobacteriaceae* were most abundant in the AI group. Notably, LEfSE analysis revealed that *Xanthomonadaceae*, *Bacillaceae*, *Brucellaceae*, *Paenibacillaceae*, and *Weeksellaceae* were most abundant in AI group. To the best of our knowledge, this is the first report to show that these bacteria were more common in acute ischemic stroke patients than controls. KEGG analysis revealed a cluster of affected metabolic modules in AI group, including folate biosynthesis, photosynthesis, the citrate cycle (TCA cycle), galactose metabolism, amino sugar and nucleotide sugar metabolism, and fructose and mannose metabolism. Stroke alters the gut microbiotic composition; conversely, microbiotic dysbiosis substantially affects stroke outcomes by modulating the immune response and the metabolic system. Intestinal microorganisms affect host metabolism and immune status, in turn modulating neuronal pathways of the enteric and central nervous systems. Intestinal probiotics may protect against stroke.

*Verrucomicrobia, Synergistetes*, and *Proteobacteria* levels were significantly higher in PI than the other groups. *Lactobacillus* levels were highest in PI group; these bacteria help to maintain good health and optimal immune function. Moreover, *Lactobacillus* exerted protective effects in rat models of cerebral ischemic stroke, inhibiting neural cell apoptosis, decreasing the cerebral infarction volume, reducing oxidative stress, and restoring neurobehavioral impairment (Chen et al., 2019). We thus speculate that *Lactobacillus* may aid recovery after stroke.

Together, the gut and brain control neurodevelopment, neurotransmitter production, and microglial function, thus modulating cerebral biochemistry and behavior (Moschopoulos and Kratimenos, 2018; Blacher et al., 2019). *Clostridium butyricum* alleviated ischemia stroke injury in diabetic mice by regulating the gut microbiota (Sun et al., 2016). Butyric acid significantly enhanced the alpha diversity of gut microbiota and reduced the levels of pathogenic bacteria such as *Bacteroides*. Conversely, brain lesions of various etiologies change the composition of the microbiota. Microbiotic manipulation may open new therapeutic approaches to various neurological diseases.

We go beyond a simple description of gut microbiotic changes in ischemic stroke patients; we propose that the gut microbiota contains non-invasive markers of early ischemic stroke. Thus, we performed fivefold cross-validation using a Random Forest model. Two species allowed of optimal LI detection: *Lachnospiraceae* (OTU_45), and *Bacteroides* (OTU_4); the AUCs under the ROCs were 0.881 and 0.872 respectively. Two species optimally detected AI: *Bilophila* (OTU_330) and *Lachnospiraceae* (OTU_338); the AUCs under the ROCs were 0.985 and 0.929 respectively. Three species optimally detected PI: *Pseudomonas* (OTU_35), *Sphingomonadaceae* (OTU_303), and *Akkermansia* (OTU_9); the AUCs under the ROCs were 1, 0.897, and 0.846 respectively. To the best of our knowledge, the microbiotic markers mentioned above have not previously been used to predict ischemic stroke.

The main limitation of the study is the small samples size, which is conduce to some potential bacteria serving as useful predictors of stroke may not be discovered. Moreover, patients with non-lacunar acute ischemic infarctions were not further subdivided into subpopulations because of the small sample size. In this condition, it is difficult to define some bacterial flora is associated with a particular subtypes of acute ischemic infarction.

We analyzed the gut microbiota of ischemic stroke patients. The microbiotas differed from those of healthy controls. Fecal microbial markers (OTUs) may be developed as useful utility when diagnosing various types of ischemic stroke, and may facilitate early appropriate therapy.

## Data Availability Statement

The raw data supporting the conclusions of this article will be made available by the authors, without undue reservation.

## Ethics Statement

The studies involving human participants were reviewed and approved by the Ethics Committee of the First Affiliated Hospital of Guangdong Pharmaceutical University. The patients/participants provided their written informed consent to participate in this study. Written informed consent was obtained from the individual(s) for the publication of any potentially identifiable images or data included in this article.

## Author Contributions

LX, YFL, and LL designed the study, collected samples, compiled the data, drafted the manuscript, and provided critical revision. YLL, WZ, JD,YG, MS, and XY compiled the data and performed the statistical analysis and data interpretation. ML collected samples and compiled the data. HL provided critical revision. XC, FL, and XX designed this study, collected samples, drafted the manuscript, and provided critical revision. All authors contributed to the article and approved the submitted version.

## Funding

This research was supported by the National Natural Science Foundation of China (grant no. 81804030).

## Conflict of Interest

The authors declare that the research was conducted in the absence of any commercial or financial relationships that could be construed as a potential conflict of interest.
